# Comparison of United States and Canadian Glaucoma Medication Costs and Price Change from 2006 to 2013

**DOI:** 10.1155/2015/547960

**Published:** 2015-04-01

**Authors:** Matthew B. Schlenker, Graham E. Trope, Yvonne M. Buys

**Affiliations:** ^1^Department of Ophthalmology & Vision Sciences, University of Toronto, 340 College Street, Suite 400, Toronto, ON, Canada M5T 3A9; ^2^Toronto Western Hospital, East Wing 6-405, Bathurst Street, Toronto, ON, Canada M5T 2S8; ^3^Kensington Eye Institute, 340 College Street, Suite 400, Toronto, ON, Canada M5T 3A9

## Abstract

*Objective*. Compare glaucoma medication costs between the United States (USA) and Canada.* Methods*. We modelled glaucoma brand name and generic medication annual costs in the USA and Canada based on October 2013 Costco prices and previously reported bottle overfill rates, drops per mL, and wastage adjustment. We also calculated real wholesale price changes from 2006 to 2013 based on the Average Wholesale Price (USA) and the Ontario Drug Benefit Price (Canada).* Results*. US brand name medication costs were on average 4x more than Canadian medication costs (range: 1.9x–6.9x), averaging a cost difference of $859 annually. US generic costs were on average the same as Canadian costs, though variation exists. US brand name wholesale prices increased from 2006 to 2013 more than Canadian prices (US range: 29%–349%; Canadian range: 9%–16%). US generic wholesale prices increased modestly (US range: −23%–58%), and Canadian wholesale prices decreased (Canadian range: −38%–0%).* Conclusions*. US brand name glaucoma medications are more expensive than Canadian medications, though generic costs are similar (with some variation). The real prices of brand name medications increased more in the USA than in Canada. Generic price changes were more modest, with real prices actually decreasing in Canada.

## 1. Introduction

Over 60 million people have glaucoma worldwide, which will likely increase to 80 million people by 2020 [1]. In the United States (USA) this disease leads to direct costs and productivity losses of $2.86 billion yearly ('01 dollars) [2]. In an effort to halt disease progression, most patients are treated with topical medications, many with laser trabeculoplasty, and some require surgical intervention.

Annual direct costs of glaucoma treatment range from $623 to $2,511 ('01 dollars) depending on disease severity. Medications represent the largest proportion of the costs (24–61%) [3]. These medication expenditures represent a significant burden to society, driving up insurance costs, taxing limited government resources, and burdening patients. The advent of topical medications has revolutionized glaucoma treatment, though medication costs affect access and compliance [4–6]. Medication noncompliance can accelerate vision loss and lead to risky and costly surgical interventions [7].

To the best of our knowledge, prices of topical glaucoma brand name and generic therapy have not been compared between the USA and Canada. Benchmarking the costs of topical glaucoma medications in two neighbouring countries with different health care systems, insurance systems, and reimbursement models will help governments and policy makers better understand the effect of the country-specific systems on costs. This study presents retail Costco prices of brand name and generic medications in both countries, models annual cost of these medications relative to one another, and models annual cost of brand name versus generic medications in each country and compares 2006 to 2013 real wholesale prices by medication and country.

## 2. Methods

### 2.1. Retail Medication Prices

One of us (M. B. Schlenker) obtained the retail noninsurance price of the commonly prescribed brand name and generic medications from Costco locations in the USA (Massachusetts) and Canada (Ontario) in October 2013. A Costco membership is not required to fill prescriptions at Costco. We cross-checked prices in other jurisdictions to ensure that our analysis was reasonably representative of each country. In the USA we cross-checked prices in Costco retail locations in Illinois and California and Costco.com, which offers delivery to any state. In Canada we cross-checked wholesale prices in Alberta and Quebec. Taxes were not included in the analysis because they are not collected on prescription medications in Canada and in most USA states [8, 9]. Throughout the analysis cash prices were utilized. Coupon discounts, public insurance coverage, copayments, private insurance coverage, and member reward benefits were excluded. For bottle sizes we used 10 mL for twice daily dosing regimens and 5 mL for once daily dosing regimens and assumed bilateral treatment. A 5 mL bottle of Xalatan (Pfizer, Inc., New York, New York, USA, and Pfizer Canada, Inc., Kirkland, Quebec, Canada) or latanoprost is not available so two 2.5 mL bottles were used. We only present data for one bottle size due to previous observations of a relatively linear relationship between cost and bottle size (with modest variation due to differences in overfill rates and modest cost savings per mL for larger bottle sizes to account for the fixed cost of the dispensing fee) [10, 11]. Costco did not carry some of the older glaucoma topical medications (e.g., istalol, metipranolol, carteolol, unoprostone, aproclonidine, echothiophate, demecarium, or pilocarpine), however these medications are rarely prescribed currently for routine glaucoma management [12]. Agents which are only available in one country were also not included (e.g., Simbrinza [Alcon, Inc., Fort Worth, Texas, USA, and Alcon, Inc., Mississauga, Ontario, Canada] is only available in the USA and Azarga [Alcon] is only available in Canada).

### 2.2. Annual Medication Cost

We utilized overfill rates and drops per mL when available based on previous reports [10, 11]. For generic medications we assumed the overfill rates and drops per mL of the original brand name medication. We did not have data for Combigan (Allergan, Inc., Irvine, California, USA, and Allergan, Inc., Unionville, Ontario, Canada) or the 0.25% beta-blocker alternatives, so the data for Alphagan P (Allergan) and 0.5% beta-blocker counterparts, respectively, were utilized. We assumed 1 drop per eye daily for prostaglandins and timolol gel and 2 drops per eye daily for the rest. A constant adjustment factor of 1.21 was assumed for misadministration of medication and noncompliance in accordance with past research [12–14].

### 2.3. Price Comparison by Country

We compared the modelled annual medication cost by expressing the more expensive Costco bottle price as a multiple of the less expensive Costco bottle price. When the US medication was more expensive the multiple was positive, and when the Canadian medication was more expensive the multiple was negative. We also calculated the additional yearly cost of the medication in the USA by taking the difference between the annual medication costs in the two countries. No adjustment was made for fluctuating exchange rates, which was on average 0.966 $Canadian : $US in October 2013 [15].

### 2.4. Price Comparison Brand versus Generic

We expressed the modelled annual medication cost of the brand name medication as a multiple of the generic medication for each country. We also calculated the additional yearly cost of the brand name medication by taking the difference between the annual medication costs of the brand name minus the generic.

### 2.5. Price Change

We obtained the wholesale medication prices in the USA and Canada (Ontario), namely, the Average Wholesale Price (AWP) in the USA and the Ontario Drug Benefit Price (ODBP) in Canada for 2006 and 2013 [16–18]. We adjusted the 2006 prices for inflation (13.2% in Canada and 15.6% in the USA from 2006 to 2013) and calculated the percentage increase or decrease of the 2013 prices relative to the inflation adjusted 2006 prices [19, 20]. Similar analyses were published in 2003 and 2008 which compared prices from 1999, 2002, and 2006 (not inflation adjusted) [10, 11]. All analyses were performed using Microsoft Excel 2013 (Microsoft Inc., Redmond, WA).

## 3. Results

### 3.1. US Annual Medication Costs

The annual US glaucoma topical medication cost ranged from $71.13 (timolol 0.25%) to $1,548.26 (Alphagan P [Allergan]) (Table 1). The average brand name medication cost was $1,165.65. Combigan (Allergan) ($1,564.30) and Alphagan P (Allergan) ($1,548.26) were the most expensive brand name medications and Trusopt (Merck & Co., Inc., Whitehouse Station, New Jersey, USA, and Merck Frosst Canada & Co., Kirkland, Quebec, Canada) ($657.95) and Azopt (Alcon) ($888.67) were the least expensive brand name medications in the study. The average generic medication cost was $281.95. Brimonidine 0.15% (Sandoz, Inc., Princeton, New Jersey, USA, and Apotex, Inc., Toronto, Ontario, Canada) ($1,280.17) was the most expensive generic medication, and timolol 0.25% ($71.13) and 0.5% ($86.06) were the least expensive medications.

### 3.2. Canadian Annual Medication Costs

The annual Canadian glaucoma topical medication costs ranged from $86.06 (timolol 0.25%) to $514.48 (Cosopt [Merck]) (Table 1). The average brand name medication cost was $306.76. Cosopt (Merck) ($514.48) was the most expensive brand name medication. The least expensive brand name medications included Betoptic S (Alcon), Timoptic XE (Valeant, Inc., Bridgewater, New Jersey, USA, and Merck), Alphagan P (Allergan), and Azopt (Alcon), all less than $250 annually. The average generic medication cost was $143.44. Dorzolamide/timolol (Sandoz and Apotex) ($374.59) was the most expensive generic medication and the least expensive generic medications included the beta-blockers and brimonidine 0.2%, all less than $115.

### 3.3. Annual Cost Comparison

**Figure 1:**
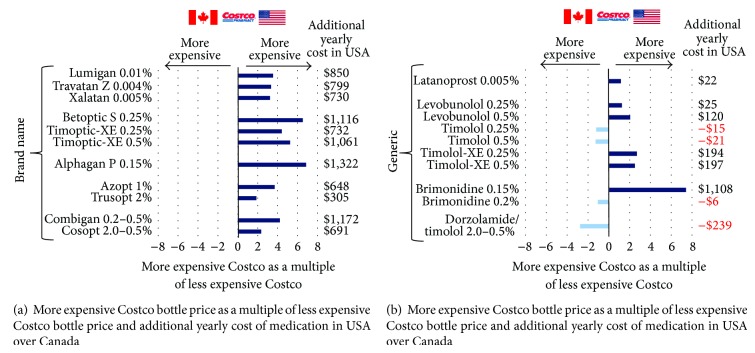
The *y*-axis first lists brand name medications (a) and then generic medications (b). The *x*-axis is the more expensive Costco bottle price as a multiple of less expensive Costco bottle price for each medication. A value of 0 (dashed line) indicates that the cost of the medication in both countries is the same. A positive value indicates that the medication in the USA is more expensive than that in Canada, and a negative value indicates that the medication in Canada is more expensive than that in the USA. For instance, Lumigan is 3.5x more expensive in the USA than in Canada. The $ value after each bar represents the additional yearly cost of the medication in the USA over Canada (negative means that medication was more expensive in Canada).

The average additional yearly cost of US medications was $858.90 for brand name medications and $138.50 for generic medications (Table 1). All the brand name medications were more expensive in the US than in Canada, ranging from 1.9 (Trusopt [Merck]) to 6.9x more expensive (Alphagan P [Allergan]) (mean = 4.1x) (Figure 1(a)). For generic medications, six were more expensive in the USA and four were more expensive in Canada, ranging from 7.4x more expensive in the USA (brimonidine 0.15%) to 2.8x more expensive in Canada (dorzolamide/timolol [Sandoz and Apotex]) (mean = 1.1x) (Figure 1(b)).

### 3.4. Generalizability of Cost Comparison

Cross-checking US Costco prices in Massachusetts to prices in Illinois, California, and on Costco.com found relative correspondence. Prices were found to be within 10% of one another, with the exception of some of the least expensive medications, generic beta-blockers, and brimonidine 0.2%, which were all within $10 of each other per bottle. AWP for generic medications does vary slightly by supplier [18]. Comparing prices in Ontario to prices in Alberta and Quebec revealed that prices were often exactly the same or within 10% with a few exceptions: timoptic XE (Valeant and Merck) cost 16% less in Quebec, Alphagan P (Allergan) cost 40% more in Alberta, and dorzolamide/timoptic (Sandoz and Apotex) cost 50% less in Quebec. While Costco prices showed regional correspondence, it should be noted that Costco is a low cost provider that not all patients may have access to, which may not offer value added services some patients require. Dispensing fees and markups can vary greatly by pharmacy [21]. For instance, in Ontario Costco's dispensing fee was $3.89 at the time of this study, while Shoppers Drug Mart's was $11.99.

### 3.5. Brand versus Generic Cost Comparison

**Figure 2:**
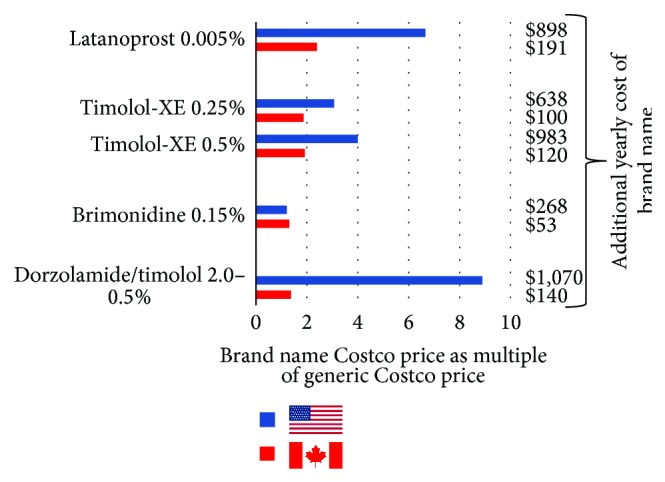
Costco brand name glaucoma medication bottle prices as a multiple of Costco generic bottle prices in USA and Canada and additional yearly cost of brand name medication over generic medication. The *y*-axis lists medications that had both a brand name and a generic alternative. The *x*-axis is the generic Costco bottle price expressed as a multiple of the brand name bottle price by country. The $ value after each bar represents the additional yearly cost of the brand name over the generic.

In the USA brand name medications cost 1.2 to 8.9x more than the generic alternatives, costing an additional $268.09 to $1,069.70 annually (Figure 2). In Canada brand name medications cost 1.3 to 2.4x more, costing an additional $53.32 to $190.73 annually over generic alternatives.

### 3.6. Price Change from 2006 to 2013

The US brand name medications real prices grew from 29% to 349% from 2006 to 2013 (mean = 101%) (Table 2). In Canada the brand name real price change ranged from −9% to 16% (mean = −1%). The US generic medications real price change ranged from −23% to 58% (mean = 12%), and the Canadian generic medication real price change ranged from −38% to 0% (mean = −22%). Only four Canadian medications' real prices increased from 2006 to 2013, including no generic medications.

## 4. Discussion

US brand name glaucoma medications cost 4x more on average than Canadian brand name glaucoma medications, representing an additional cost of $305 to $1,322 annually per patient per drug for US drugs over Canadian drugs. US and Canadian generic costs are more similar, though variation exists (range: Canadian drug 2.8x more expensive to US drug 7.4x more expensive). US brand name medication real prices increased significantly from 2006 to 2013 (29% to 349%), while generic medication prices were more stable. Overall Canadian medications saw a small decrease in real prices over the study period, more so for generic than for brand name medications.

This study highlights the wide variability in costs of glaucoma topical medication by drug and country and identifies many trends clinicians that should be aware of as they prescribe medications to patients. For US clinicians, some brand name medications cost up to $1,000 more annually than other brand name medications. Comparing US brand name to generic medication cost, generic medications cost on average $771 less annually than brand name medications. For prostaglandin analogs—many clinicians' first line agent—generic latanoprost costs approximately $900 less annually than brand name alternatives. For Canadian clinicians, brand name medications cost between $200 and $500 and are $50 to $200 more annually than generic medications.

The results of this study also provide important data for government officials, insurance companies, policy makers, and health advocates. The USA spent $329 billion in 2013 on prescription products, one of the drivers of the highest per capita health care spending in the world [22]. It is not surprising that the US's market system may lead to higher prices than found in Canada, because in Canada patented medication prices are regulated by the Patent Medicines Prices Review Board (PMPRB). The PMPRB ensures that prices are set at the median price from the following countries: France, Germany, Italy, Sweden, Switzerland, the United Kingdom, and the USA [23]. New entry drug prices are set relative to the cost of the current therapies, and existing patented medications cannot increase more than the Consumer Price Index [23]. In the USA prices are not regulated, but rather subject to market forces and insurance reimbursement policies. Pharmaceutical companies resist price controls because their resultant profits may not reflect the research and development that went into a drug or the fact that most drugs researched do not become approved medications [24].

In contrast to brand name medication pricing dynamics, several reports have suggested that generic medication prices in Canada are among the highest in the world due to government price setting (relative to brand name prices) not allowing for appropriate competition [25–27]. Ontario is transitioning to a policy of generic medication reimbursements set at twenty-five percent of the brand name equivalent for solid forms and thirty-five percent for nonsolid forms [28]. Fixed percentage reimbursements can serve as a ceiling or a floor depending on the balance between the manufacturing cost and the market competitive dynamics. Studies have suggested that the Canadian generic price setting has been serving as a ceiling, resulting in a clustering of pricing around maximum allowable levels [29]. Of note these studies do not focus on glaucoma topical medications [30]. In this study we report that Canadian generic glaucoma medication prices are on average similar to US generic prices, with some variation. We also observed that Canadian generic prices were decreasing in real terms, while the US prices were overall increasing (though not as much as the brand name alternatives). So, the concerns of high generic prices in Canada are not borne out in the topical glaucoma medication market.

Strengths of this analysis include being able to compare retail prices from Costco, which is a nationwide chain in both countries and a low cost provider in both countries. Then, modelling the prices from price per bottle into annual costs provides practical information for clinicians, patients, and policy makers. For the price change analysis over time, we believe that looking at real prices is more useful than nominal prices and hope that this approach is replicated in further research.

In terms of limitations, while this study measures cost, it does not measure cost effectiveness. Efficacy, side effect profile, and tolerability must be weighed in the context of a patient's individual situation for day-to-day clinical decision making. As well, the price data is not representative of what all patients in the respective countries will pay. The price comparison by region was not exhaustive, so the presented prices may not hold true for different regions in the different countries. More importantly, costs can vary significantly by pharmacy due to differences in dispensing fees and retail markups. As well, this analysis does not adjust for coupon discounts, public insurance coverage, copayments, private insurance coverage, or member reward benefits.

Another limitation is that the overfill rates and drops per mL were not available for all medications, may have changed for medications, may not be updated if the bottle or medication formulation has changed, and are difficult to measure. Several assumptions had to be made regarding these values. High overfill rates and drops per mL would decrease the annual cost of medications and vice versa. Even different makers of the same glaucoma medication may also have different drop sizes or overfill rates [31]. Of course, if the overfill rate and drops per mL were constant across manufacturers in both Canada and the USA, any incorrect assumptions would cancel out in the comparative analyses. Another limitation is that this analysis only represents a snap shot in time. Fluctuating exchange rates, macroeconomic trends, and shifting political policies can significantly affect future prices. There has been talk of policies related to the Affordable Care Act affecting US generic prices. Repeat analysis should be performed in the future to assess new trends, with this study and previous studies serving as baselines [10, 11].

In summary, in October 2013 Costco brand name glaucoma topical medications cost significantly more in the USA than in Canada, while generic costs were similar in the two countries. US wholesale brand name medication real prices increased significantly from 2006 to 2013, while generic real price increases were more modest. In Canada most topical glaucoma medication prices fell in real terms. These results are an important baseline for clinicians, government officials, insurance companies, policy makers, and health advocates.

## Figures and Tables

**Table 1:** Retail cash price cost comparison of glaucoma topical medications in the USA and Canada.

Product name	Active ingredient	Conc. %	Size mL	Price per bottle		Measured volume^1^ mL	Drops per mL^1^	Bottles per year	Wastage adjustment^2^	Yearly cost		Additional yearly cost in USA $
				Costco in USA $US	Costco in Canada $Cdn					Costco in USA $US	Costco in Canada $Cdn	
Prostaglandins												
Brand												
Lumigan	Bimatoprost	0.01	5	228.69	64.61	5.29	32.23	4.3	1.21	1,184.78	334.70	850.08
Travatan Z	Travoprost	0.004	5	218.19	65.35	5.03	33.58	4.3	1.21	1,141.02	341.74	799.29
Xalatan^3^	Latanoprost	0.005	5	222.24	68.80	6.26	29.66	3.9	1.21	1,057.27	327.31	729.96
Generic												
Latanoprost^4^	Latanoprost	0.005	5	33.40	28.71	6.26	29.66	3.9	1.21	158.89	136.58	22.32
*β*-Blockers												
Brand												
Betoptic S	Betaxolol	0.25	10	191.74	29.40	9.76	26.34	5.7	1.21	1,317.61	202.03	1,115.58
Timoptic-XE^5^	Timolol	0.25	5	120.61	27.30	5.21	21.62	6.5	1.21	945.80	214.10	731.70
Timoptic-XE	Timolol	0.5	5	167.21	31.90	5.21	21.62	6.5	1.21	1,311.22	250.18	1,061.05
Generic												
Levobunolol^5^	Levobunolol	0.25	10	17.71	14.08	10.01	25.71	5.7	1.21	121.57	96.67	24.90
Levobunolol	Levobunolol	0.5	10	33.96	16.46	10.01	25.71	5.7	1.21	233.11	113.02	120.10
Timolol^5^	Timolol	0.25	10	11.95	14.46	10.03	29.59	4.9	1.21	71.13	86.06	−14.93
Timolol	Timolol	0.5	10	13.57	17.15	10.03	29.59	4.9	1.21	80.77	102.10	−21.32
Timolol-XE^5^	Timolol-XE	0.25	5	39.31	14.57	5.21	21.62	6.5	1.21	308.26	114.25	194.01
Timolol-XE	Timolol-XE	0.5	5	41.83	16.67	5.21	21.62	6.5	1.21	328.02	130.69	197.33
*α*2-Agonists												
Brand												
Alphagan P	Brimonidine	0.15	10	204.67	29.86	10.37	22.52	6.3	1.21	1,548.26	225.86	1,322.40
Generic												
Brimonidine^4^	Brimonidine	0.15	10	169.23	22.81	10.37	22.52	6.3	1.21	1,280.17	172.54	1,107.63
Brimonidine	Brimonidine	0.2	10	15.59	16.50	10.28	26.28	5.4	1.21	101.94	107.91	−5.97
CAIs												
Brand												
Azopt	Brinzolamide	1.0	10	148.94	40.32	10.04	29.49	4.9	1.21	888.67	240.57	648.10
Trusopt	Dorzolamide	2.0	10	87.46	46.87	9.98	23.53	6.2	1.21	657.95	352.61	305.35
Combination												
Brand												
Combigan^6^	Brimonidine/timolol	0.2–0.5	10	206.79	49.01	10.37	22.52	6.3	1.21	1,564.30	370.76	1,193.55
Cosopt	Dorzolamide/timolol	2.0–0.5	10	162.25	69.26	10.65	22.33	6.1	1.21	1,205.27	514.48	690.80
Generic												
Dorzolamide/timolol^4^	Dorzolamide/timolol	2.0–0.5	10	18.25	50.43	10.65	22.33	6.1	1.21	135.57	374.59	−239.02

^1^Fiscella et al. [10] and Rylander et al. [11].

^2^Iordanous et al. [13], Platt et al. [14], and Lee and Hutnik [12].

^3^Only 2.5 mL bottle available so price for 5 mL is 2.5 mL × 2.

^4^Assumed equivalent brand name's measured volume and drops per mL.

^5^Assumed equivalent 0.5%'s measured volume and drops per mL.

^6^Assumed Alphagan P's measured volume and drops per mL.

**Table 2:** Glaucoma topical medications price change from 2006 to 2013 in the USA and Canada.

Product name	Active ingredient	Conc. %	2006 unit price		2006 unit price in 2013 dollars^3^		2013 unit price		Inflation adjusted % change	
			AWP^1^	ODBP^2^	AWP^1^	ODBP^2^	AWP^1^	ODBP^2^	AWP^1^	ODBP^2^
			$US	$Cdn	$US	$Cdn	$US	$Cdn	%	%
Brand										
Prostaglandins										
Lumigan	Bimatoprost	0.01	26.79	10.40	30.97	11.77	50.25	11.12	62%	**−6%**
Travatan Z	Travoprost	0.004	23.88	10.60	27.61	12.00	46.25	11.26	68%	**−6%**
Xalatan	Latanoprost	0.005	23.54	10.40	27.21	11.77	52.44	11.18	93%	**−5%**
*β*-Blockers										
Betoptic S	Betaxolol	0.25	9.20	2.23	10.64	2.52	20.66	2.34	94%	**−7%**
Timoptic-XE	Timolol	0.25	—	3.26	—	3.69	31.80	4.29	—	16%
Timoptic-XE	Timolol	0.5	6.72	3.90	7.77	4.41	34.85	5.13	349%	16%
*α*2-Agonists										
Alphagan P	Brimonidine	0.15	9.32	2.31	10.77	2.61	22.41	2.38	108%	**−9%**
Carbonic anhydrase inhibitors										
Azopt	Brinzolamide	1.0	7.56	3.22	8.74	3.65	15.69	3.34	80%	**−8%**
Trusopt	Dorzolamide	2.0	6.18	3.30	7.14	3.74	9.20	3.94	29%	5%
Combination										
Combigan	Brimonidine/timolol	0.2–0.5	—	3.94	—	4.46	22.50	4.13	—	**−7%**
Cosopt	Dorzolamide/timolol	2.0–0.5	11.33	5.00	13.10	5.66	16.89	5.99	29%	**6%**
Generic^4^										
Prostaglandins										
Latanoprost	Latanoprost	0.005	—	—	—	—	22.05	3.83	—	—
*β*-Blockers										
Levobunolol	Levobunolol	0.25	2.93	0.93	3.38	1.06	2.72	0.93	−20%	**−12%**
Levobunolol	Levobunolol	0.5	3.23	1.23	3.73	1.40	4.08	1.15	9%	**−18%**
Timolol	Timolol	0.25	2.35	1.28	2.71	1.45	2.76	0.97	2%	**−33%**
Timolol	Timolol	0.5	3.23	1.51	3.74	1.71	2.88	1.21	**−23%**	**−29%**
Timolol-XE	Timolol-XE	0.25	5.42	—	6.26	—	9.90	1.96	58%	—
Timolol-XE	Timolol-XE	0.5	6.44	—	7.44	—	11.70	2.34	57%	—
*α*2-Agonists										
Brimonidine	Brimonidine	0.15	—	—	—	—	20.17	1.73	—	—
Brimonidine	Brimonidine	0.2	6.52	1.65	7.54	1.87	8.27	1.16	10%	−38%
Combination										
Dorzolamide/timolol	Dorzolamide/timolol	2.0–0.5	—	—	—	—	10.88	4.26	—	—

^1^Average Wholesale Price from Red Book.

^2^Ontario Drug Benefit Price.

^3^Inflation from 2006 to 2013 estimated to be 15.6% for USA and 13.2% for Canada.

^4^Generic price is an average of all generic companies offering the medication.
